# Dairy and Wine Industry Effluents as Alternative Media for the Production of *Bacillus*-Based Biocontrol Agents

**DOI:** 10.3390/bioengineering9110663

**Published:** 2022-11-08

**Authors:** Selena Dmitrović, Ivana Pajčin, Vanja Vlajkov, Mila Grahovac, Aleksandar Jokić, Jovana Grahovac

**Affiliations:** 1 Faculty of Technology Novi Sad, University of Novi Sad, Bulevar Cara Lazara 1, 21000 Novi Sad, Serbia; 2 Faculty of Agriculture, University of Novi Sad, Trg Dositeja Obradovića 8, 21000 Novi Sad, Serbia

**Keywords:** aflatoxin B1, biological control, fructose, glucose, Gompertz equation, lactose, logistic equation, Luedeking-Piret equation, microbial biomass, lipopeptide

## Abstract

Food industry effluents represent one of the major concerns when it comes to environmental impact; hence, their valorization through different chemical and biological routes has been suggested as a possible solution. The vast amount of organic and inorganic nutrients present in food industry effluents makes them suitable substrates for microbial growth. This study suggests two valorization routes for whey as dairy industry effluent and flotation wastewater from the wine industry through microbial conversion to biocontrol agents as value-added products. Cultivations of the biocontrol strain *Bacillus* sp. BioSol021 were performed in a 16 L bioreactor to monitor the bioprocess course and investigate bioprocess kinetics in terms of microbial growth, sugar substrate consumption and surfactin synthesis, as an antimicrobial lipopeptide. The produced biocontrol agents showed high levels of biocontrol activity against mycotoxigenic strains of *Aspergillus flavus*, followed by a significant reduction of sugar load of the investigated effluents by the producing microorganisms. With proven high potential of whey and winery flotation wastewater to be used as substrates for microbial growth, this study provides grounds for further optimization of the suggested valorization routes, mostly in terms of bioprocess conditions to achieve maximal techno-economical feasibility, energy saving and maximal reduction of effluents’ organic and inorganic burden.

## 1. Introduction

### 1.1. Biological Control as a Possible Route for Increased Sustainability of Agricultural Production

The global issue that has resulted in significant economic losses, measured in billions of U.S. dollars, is attributed to plant diseases caused by fungal pathogens [1]. Regarding *Aspergillus flavus* in this role, the problem is even more concerning considering that the consequences of plant mycotoxin contamination could influence human health. *Aspergillus flavus* is a well-known opportunistic pathogen of many crops of economic interest, including maize, peanuts and cotton, and a major producer of aflatoxins. According to classification by the International Agency for Research on Cancer (IARC), aflatoxin B1 (AFB1) is classified in the 1a group of carcinogenic compounds for humans and animals [2,3,4]. AFB1 is defined as the most potent natural carcinogen and one of only a few mycotoxins that have been used in the production of biological weapons [5].

Increasing global awareness on the negative consequences of chemical product use for plant protection has led to a growing interest in biopesticides among both agricultural producers and the general public. Additional pressures are supported by changes in legislation, favoring the application of ecological alternatives [6]. The use of biopesticides in plant protection is recognized as a promising solution with a number of positive outcomes, with the emphasis on the reduction of the amount of pesticide residues in food, resulting in a reduction of their negative impacts on people as end consumers [7]. Microbial-based biopesticides have shown great potential in the suppression of plant pathogens, as highly effective, selective and environmentally friendly solutions [8,9]. Almost 90% of the total biopesticide market consists of products based on microorganisms [10]. The development of microbial-based products is directly influenced by the availability of a potent isolate as a candidate for the suppression of certain plant pathogens, which is considered a far more specific approach compared to the products of chemical origin for the same purpose. Other than the primary effect of antagonistic activity, a number of microorganisms have additional properties that have positive impacts on plants, such as growth promotion and improved nutrition, provided by stimulating the uptake of micro and macro elements available in the soil [8]. Bacterial biopesticides with representatives of the *Bacillus* genus as active components express intensive activity in the plant-environment-pathogen relationship, through multiple mechanisms of action including plant growth promotion (PGP), induction of systemic plant resistance, biofilm formation, competition for nutrients and space, lytic effect and antibiotic production [11]. One of the most important mechanisms of the *Bacillus* biocontrol strains relies on the production of a wide spectra of metabolites with antibacterial/antifungal activity against the target plant pathogens. Among the important metabolites synthesized by *Bacillus* species, lipopeptides are usually emphasized, with the greatest focus on surfactin, iturin and fengycin families [12]. Surfactins are a group of lipopeptide microbial surfactants which, other than antimicrobial activity, have widespread application in various industrial fields, including oil recovery, biopesticides and food processing, as well as the cosmetic and pharmaceutical industries [13]. The family of surfactins is well known for great structural diversity due to the type of amino acids in the peptide chain and the length and isomery of the lipidic chain. The impressive biodiversity of these compounds mainly results from their biosynthetic mechanisms [14]. Although the biosynthetic pathway and the nature of the multienzyme systems involved in the synthesis of surfactins has not been entirely established, it has been discovered that surfactins and other bioactive lipopeptides are non-ribosomally synthesized by their producer organisms [15]. The direct antagonism of surfactins against a wide spectrum of plant pathogens has been previously confirmed [16,17], with additional *in planta* benefit based on their mediator role in facilitating plant colonization by the biocontrol and PGP *Bacillus* strains [18]. Hence, the *Bacillus*-based biocontrol products usually include biomass of the microbial active component, as well as the beneficial metabolites produced by the selected producing strain.

### 1.2. Alternative Media for Biocontrol Agents Production

Despite the potential of existing biopesticides, the number of products available on the global market is still limited. The lack of full commercialization is attributed to the high costs of biotechnological production, with an emphasis on the costs of production equipment, sterility requirements and commercially-used nutritive media. The usage of synthetic media is economically unsustainable, but on the other hand, the main advantage lies in their consistent chemically-defined composition. The answer to the existing limitations is the usage of alternative complex media based on industrial effluents, which can be beneficial from the point of view of economic efficiency of the designed bioprocess, and are additionally considered an eco-friendly solution for improved sustainability of the production processes aimed at microbial biocontrol agent production [19]. Food industry effluents are of special interest as potential raw materials for microbial biopesticides production; many studies have investigated the high organic and inorganic burden of these effluents, making their treatment procedure mandatory before disposal [20]. Furthermore, this could also present a way to recycle water and decrease industrial fresh water consumption in alignment with the principles of the circular economy and industrial symbiosis [21]. Prior to medium formulation and usage in microbial-based processes, the complexity of their composition makes it necessary to characterize the raw materials in terms of expected seasonal variations in terms of content and investigate the potential inhibitory effects on microorganism growth [22].

#### 1.2.1. Dairy Industry Effluents: A Brief Overview

The dairy industry occupies an important position among the other food industry branches as it meets the needs of the global population with nutritionally rich products included in the everyday diet, such as milk, cheese, butter, ghee, milk powder, etc. At the same time, the dairy industry is responsible for generating a significant amount of pollution due to its heavy use of water in the production process and the heavy emission of effluents into the environment [23]. In Europe alone, nearly 29 million tons of dairy products end up as waste every year [23]. Cheese production generates three different waste/byproduct streams, including cheese whey (CW), secondary CW and dairy wastewater [24]. Although it could be used as a valuable byproduct of the dairy industry, CW is one of the most striking industry effluents that threatens the environment and human health. CW is a green-yellow by-product of cheese or casein production, with an estimated worldwide production of approximately 190 billion kg/year [25]. In general, CW accounts for about 90% of milk volume, retains 55% of milk nutrients and 20% of milk proteins [25]. The excessive amount of whey (approximately 9 L from 1 kg of cheese production), released during the production process, causes a concerning environmental problem [26]. Dairy effluents are characterized by high COD (chemical oxygen demand) and BOD (biological oxygen demand) levels due to the milk proteins and lactose they contain [26]. Concentrations of COD and BOD in CW are usually in the range of 50–102 g/L and 27–60 g/L, respectively, and the BOD/COD ratio is normally above 0.5, which constitutes a substrate easily biodegradable by anaerobic or aerobic digestions [27]. The treatment of whey and other dairy products can be incomplete in the aerobic treatment system or is difficult to achieve in the anaerobic system due to high COD content and a tendency for rapid acidification [26]. The whey pH value depends on the CW type (acidic or sweet). The acidic CW has a pH value lower than 5; it is obtained after fermentation or the addition of organic acids, while the sweet CW, which is obtained by the addition of proteolytic enzymes, such as chymosin, has a pH value between 6 and 7 [27]. The acidic CW has a higher ash and lower protein content than the sweet CW. CW contains sodium, potassium and calcium salts (0.46–10%), while calcium concentrations (1.2–1.6 g/L) in acidic CW are two times higher than the values observed in the sweet CW [25,27]. The high sodium concentrations can cause problems when operating biological digesters [27]. Dairy effluents can be purified, treated or used in other industries by the development of technologies such as reverse osmosis, drying, hydrolysis, ion exchange, nanofiltration, ultrafiltration and electrodialysis. On the other hand, biological methods like microbial fermentation or anaerobic digestion can be more sustainable than traditional methods, and produce more valuable compounds. Bioconversion of whey and other dairy effluents into value-added products is an essential solution in the modern era. Dairy industry by-products are a great source of lactose and other nutrients that can support microbial growth [26]. The integration of dairy industry effluents into the biorefinery context will make a significant contribution to the development of a wider palette of bio-products aligned with the principles of a circular economy, providing a significant boost to further bioeconomy development. Some of the proposed valorization routes have suggested the feasible use of dairy by-products/effluents for the microbial production of edible products, such as lactic acid, or in microbial biomass production [26]. 

#### 1.2.2. Wine Industry Effluents: A Brief Overview

Grapes, as one of the most consumed fruit crops, are widely and globally cultivated with a total output of over 78 million tons in 2020 [28]. More than 70% of the produced grape amount is used for winemaking [28,29]. Wine production effluents are being generated in large amounts, accounting for around 20% of the total grape mass, including solid effluents such as seeds, skins, stems and residual pulp, as well as liquid effluents, mostly in the form of wastewater from different wine production stages [28]. Approximately 1.3–1.5 kg of solid waste is being generated during the production of one liter of wine, with a water content of 65–75% [30], while on the other hand, 2–14 L of wastewater could be generated per one liter of the produced wine, depending on the production technology [31]. Consequently, winery waste management is becoming a serious issue in the wine-making industry [32]. Solid winery effluents have been considered as a high value-added animal feed and organic fertilizer. However, high fibrousness and low digestibility limit their large-scale application [33]. On the other hand, winery effluents are rich in valuable compounds including oil, polyphenols and carbohydrates, which can be used in the food, pharmaceutical, cosmetic or bioenergy industries [34]. Recovery of the by-products in the wine industry is a very important process that helps to minimize ecological problems. Current technologies for the processing of wine products provide the implementation of integrated technologies, in which the main product and by-products are given similar attention by applying intensive industrialization. Modern technical and scientific achievements have allowed for the proposal of different means of waste recovery, with an exceptional economic advantage in terms of generated products, sometimes greater than that obtained from wine products. In the wine industry, a series of resulting by-products and waste can be put to good use: the clusters which are separated before pressing; the grappa resulting from pressing the grapes; the yeasts deposited on the bottom of vessels after fermentation of the wine; the tartar on the walls of the vessels when storing wine [35]. Winery wastewater is a major waste stream, resulting from a number of activities that include washing the floor, open areas, equipment, bottles, barrels and storage tanks [22,36]. The organic content of winery wastewater depends on the origin production stage, but usually includes sugars, alcohols, acids and polyphenols [37]. The presence of a high load of organic and inorganic contaminants in winery wastewater results in very high COD (320–49105 mg/L) and BOD_5_ values (203–22418 mg/L). Contrary to the application of the solid winery effluents as biofertilizers, applications of winery wastewater to soil without an appropriate monitoring and treatment program can change the physicochemical properties of groundwater by affecting its color, pH value and electrical conductivity due to the effluent’s low pH value and unfavorable C/N ratio. Since winery wastewater at certain concentrations may cause significant environmental damage if released untreated into the environment, it is very important to find the appropriate processes for its treatment. Winery effluents with a reduced environmental footprint could be obtained through different treatment procedures suggested in the literature, such as physicochemical, biological, membrane separation or advanced oxidation processes, or a combination of these [22]. Considering the wide range of microbially acceptable nutrients in winery effluents, their microbial conversion to different value-added products has been suggested as a possible treatment procedure [38].

### 1.3. Hypothesis and the Main Objective of the Research

According to the previously summarized literature, the hypothesis of this study is that the specific dairy and wine industry effluents could be applied as possible raw materials for the production of biocontrol agents based on beneficial *Bacillus* strains as value-added products obtained by valorization of the food industry effluents. The main objective of this study was an investigation of two valorization routes of the following food industry effluents: cheese whey (CW) from the dairy industry and winery flotation wastewater (WFW) from the wine industry through microbial conversion to biocontrol agents as value-added products. In order to better understand the possible application of these effluents as a medium basis for the production of microbial biocontrol agents, cultivations of the producing strain *Bacillus* sp. strain BioSol021 were performed in the 16 L lab-scale bioreactor to monitor the most important bioprocess parameters, including an investigation of bioprocess kinetics in terms of microbial growth, sugar substrate consumption (as the major contaminant in the effluents) and surfactin production as one of the desirable metabolites with proven antimicrobial and plant-beneficial activity. Along with the monitoring and investigation of cultivations, antimicrobial activity testing was simultaneously performed to test the suppression ability of the produced biocontrol agents against the aflatoxigenic *Aspergillus flavus* strains. 

## 2. Materials and Methods

### 2.1. Microorganisms

The producing microorganism used in this study was *Bacillus* sp. strain BioSol021, isolated from the rhizosphere of common beans or kidney beans (*Phaseolus vulgaris*) and identified using 16S rRNA gene sequencing (GenBank sequence accession number ON569805) and biochemical characterization using VITEK2 BCL cards (Biomerieux, Marcy-l′Étoile, France) as a member of the operational group *Bacillus amyloliquefaciens* [39]. The test microorganisms or phytopathogens used in this study were *Aspergillus flavus* strains SA2B SS and PA2D SS isolated from maize with proven aflatoxigenic potential [40]. 

The producing microorganism (*Bacillus* sp. strain BioSol021) was kept on a semi-solid commercial medium nutrient agar (HiMedia Laboratories, Thane West, Maharashtra, India). Fungal test microorganisms (*Aspergillus flavus* SA2B SS and PA2D SS) were kept on a semi-solid semi synthetic medium SMA (Sabouraud maltose agar), obtained by combining SMB (Sabouraud maltose broth, HiMedia Laboratories, Thane West, Maharashtra, India) and 2% (*w*/*v*) agar (HiMedia Laboratories, Thane West, Maharashtra, India). All microorganisms were stored at 4 °C. In order to stimulate their metabolic activity and ability to reproduce, microorganisms were refreshed by applying the same nutrient media that was used for their storage under the following conditions: *Bacillus* sp. BioSol021 at 28 °C for 48 h; *Aspergillus* spp. at 26 °C for 120 h.

### 2.2. Cultivation Media

Inoculum of the producing microorganism *Bacillus* sp. BioSol021 was prepared using the synthetic liquid medium nutrient broth (HiMedia Laboratories, Thane West, Maharashtra, India). Cultivations of the producing microorganisms were performed using media based on CW as the dairy industry effluent and WFW. The initial quality parameters of the effluents used for media preparation are given in Table 1, and the methods employed to determine these parameters are explained in Section 2.4. The pH value of the CW was adjusted to 7.0 ± 0.1 using the 1 M NaOH and the CW with the adjusted pH value was further used as a cultivation medium. The WFW was diluted in the ratio 1:3 (*v*/*v*) using the distilled water and the initial pH value of the cultivation medium was set to the same value as in the case of the CW-based medium. All media were sterilized by autoclaving (121 °C, 20 min, 2.1 bar). 

### 2.3. Inoculum Preparation and Cultivation Parameters

Preparation of the inoculum of the *Bacillus* sp. BioSol021 was performed by seeding the liquid commercial medium with loopful biomass of the previously refreshed microorganism, and by cultivation for 48 h at 28 °C with external stirring on a laboratory shaker (150 rpm) and spontaneous aeration. The amount of inoculum was 10% (*v*/*v*) of the working bioreactor volume. Cultivations of *Bacillus* sp. BioSol021 using both investigated effluent-based media lasted 96 h at 28 °C in a 16 L-laboratory bioreactor (EDF–15.4_1, A/S Biotehniskais center, Riga, Latvia) with the working volume corresponding to ⅔ of the total volume, with a stirring rate of 100 rpm using the three impeller Rushton turbine and with an aeration rate of 1.5 vvm (volume of sterile air/(volume of the medium·min)). During the cultivations, continuous in line monitoring of temperature, pH value and partial oxygen pressure (pO_2_) was performed. Offline analyses to determine dry cell weight, sugar substrate concentration, surfactin concentration and antimicrobial activity against the target phytopathogens were performed using cultivation broth samples collected at predefined time intervals. Data on the cultivation courses were visualized using the LabPlot v. 2.9 software (github.com/KDE/labplot) [41].

### 2.4. Analytical Methods

#### 2.4.1. Gravimetric Method for Biomass Dry Weight Measurement

The collected samples of the *Bacillus* sp. BioSol021 cultivation broths during the bioreactor cultivations were centrifuged (12,000 g, 10 min, 25 °C, Z 326 K, Hermle LaborTechnik, Wehingen, Germany) to separate microbial biomass from the liquid part of the cultivation broth. The biomass pellet obtained from 5 mL of the cultivation broth sample was dried (105 °C) until reaching a constant mass in three consecutive measurements. Biomass concentration of the producing microorganism *Bacillus* sp. BioSol021 (g/L) was calculated based on the measured biomass dry weight and the initial cultivation broth sample volume.

#### 2.4.2. Well-Diffusion Assay for Antimicrobial Activity Testing

Antifungal activity of *Bacillus* sp. BioSol021 cultivation broth samples was tested using the well diffusion assay against the test microorganisms *Aspergillus flavus* SA2B SS and PA2D SS. In 90 mm Petri plates, a layer of mixture of the SMA medium (15 mL) and fungal pathogen suspension in sterile saline (1 mL-10^5^ CFU/mL), homogenized using the vortex mixer, was spread. After the medium solidification, three wells per plate with a diameter of 10 mm were made. In each well, 100 μL of the cultivation broth sample was added. The incubation was performed at 26 °C for 96 h, followed by measurements of the inhibition zone diameters [39,40].

#### 2.4.3. CPC-BTB Method for Surfactin Quantification

The surfactin concentration in the *Bacillus* sp. BioSol021 culture supernatant was measured by the CPC-BTB (cetylpyridinium chloride-bromothymol blue) method [42]. The culture supernatant was obtained by centrifugation of the collected samples of cultivation broths during the bioreactor cultivations. Bromothymol blue (BTB) and the mediator cetylpyridinium chloride form a green colored complex. When surfactin is added, it forms a colorless complex with the CPC, releasing BTB molecules to the medium and generating a color shift that can be detected spectrophotometrically. Quantification was carried out by mixing 300 μL of the supernatant sample and 2.4 mL of the CPC-BTB reagent, and the mixture was kept at 25 °C for 5 min. Finally, absorbance at 600 nm was measured (UV 1800, Shimadzu, Kyoto, Japan) and the surfactin concentration was calculated by the standard curve [43].

#### 2.4.4. HPLC Method for Sugar Concentration Determination

The High-Performance Liquid Chromatography (HPLC) method was applied for the quantification of sugar substrates in the effluent-based cultivation broths: fructose, glucose and lactose. The analyses were performed using a Thermo Scientific Dionex UltiMate 3000 system (Thermo Fisher Scientific, Waltham, MA, USA) with a ZORBAX NH2 column (Agilent Technologies, Santa Clara, CA, USA) at the column temperature of 25 °C with a flow rate of 1.2 mL/min using isocratic elution with 75% acetonitrile in the HPLC water as a mobile phase. The detection was performed using a refractive detector (RefractoMax 521, Knauer, Berlin, Germany) with a detector temperature of 25 °C. All the cultivation broth samples were centrifuged as previously described, and the supernatant samples used for sugar content analysis were filtered through a 0.22 µm membrane prior to autosampler column injection (10 μL). 

### 2.5. Investigation of Bioprocess Kinetics

The bioprocess data on the producing microorganism’s biomass concentration, initial and residual sugar content in the cultivation media and surfactin concentration were used to generate kinetic models describing microbial growth, substrate consumption and product formation using non-linear regression analysis. The main microbial growth parameters (*X*_0_—initial biomass content (g/L), *X_max_*—maximal biomass content (g/L), *μ*—maximal specific growth rate (1/h)) were determined using the Gompertz (Equation (1)) and the logistic equation (Equation (2)):

(1) $$X\;\left(t\right)=X_{max}\cdot {\left(\frac{X_{0}}{X_{max}}\right)}^{e^{-\mu \cdot t}}$$

(2) $$X\left(t\right)=\frac{X_{max}\cdot X_{0}}{\left(X_{max}-X_{0}\right)\cdot e^{-\mu \cdot t}+X_{0}}$$

where *X* (g/L) is the producing microorganism’s biomass concentration in the time *t* (h). 

The modified LuedeKing-Piret equation [44] was employed to determine the kinetic factors related to sugar substrate consumption (Equations (3) and (4)) and product formation (Equations (5) and (6)):

(3) $$-\frac{dS}{dt}=\alpha \cdot \frac{dX}{dt}+\beta \cdot X$$

(4) $$S\left(t\right)=S_{0}-\alpha \cdot X_{0}\cdot \left(\frac{e^{\mu \cdot t}}{1-\frac{X_{0}}{X_{max}}\cdot \left(1-e^{\mu \cdot t}\right)}-1\right)-\beta \cdot \frac{X_{max}}{\mu }\cdot \ln(1-\frac{X_{0}}{X_{max}}\cdot \left(1-e^{\mu \cdot t}\right)$$

(5) $$\frac{dP}{dt}=\gamma \cdot \frac{dX}{dt}+\delta \cdot X$$

(6) $$P\left(t\right)=P_{0}+\gamma \cdot X_{0}\cdot \left(\frac{e^{\mu \cdot t}}{1-\frac{X_{0}}{X_{max}}\cdot \left(1-e^{\mu \cdot t}\right)}-1\right)+\delta \cdot \frac{X_{max}}{\mu }\cdot \ln(1-\frac{X_{0}}{X_{max}}\cdot \left(1-e^{\mu \cdot t}\right)$$

where *S* (g/L) is the sugar substrate concentration in the time t (h); S_0_—initial sugar substrate content (g/L); α—growth-related substrate consumption coefficient (g_substrate_/g_biomass_); *β*—maintenance coefficient (1/h); *P* (g/L) is surfactin concentration in the time t (h); *P*_0_—initial surfactin content (g/L); *γ* (g_product_/g_biomass_) and δ (1/h)—growth and non-growth related product formation coefficients. In order to assess the adequacy of the selected kinetic equations to fit the experimental data, the coefficient of determination (R^2^) was employed. Bioprocess kinetic analysis was performed using GraphPad Prism software v. 8.4.3 (GraphPad Software, San Diego, CA, USA) [45]. 

## 3. Results and Discussion

### 3.1. Bioprocess Course Monitoring—Temperature, pH Value and DO Content

**Figure 1:**
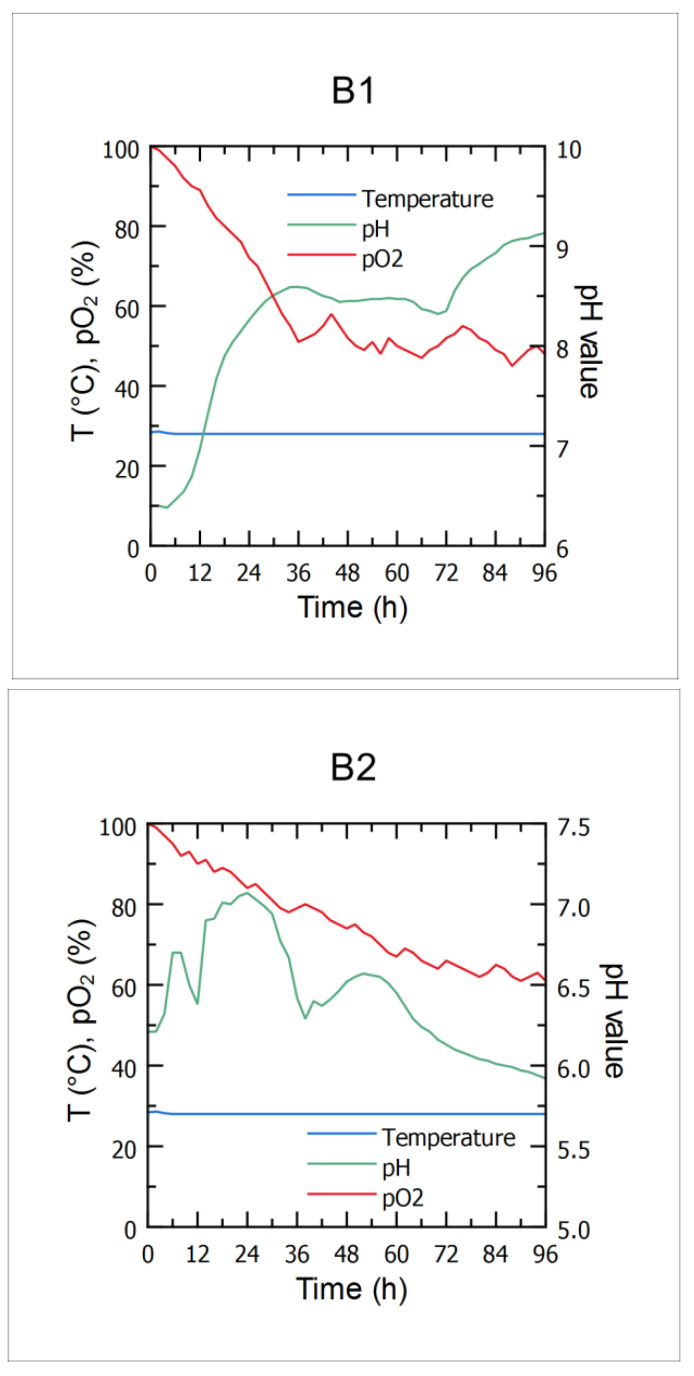
Cultivations of *Bacillus* sp. BioSol021 in a 16 L bioreactor - cultivation courses in terms of temperature, pH value and DO content using the food industry effluents-based media: B1—cheese whey from dairy industry, B2—winery flotation wastewater.

Cheese whey and winery flotation wastewater were selected as potential raw materials for biocontrol agent production according to their wide availability as food industry effluents and the literature data regarding the content of the most important nutrients suitable for microbial growth. The main criteria relevant for preparation of the cultivation media for microbial growth, when it comes to composition of the aforementioned effluents, were included during the effluent screening, including pH value (in order to check whether it should be adjusted prior to cultivation of the producing *Bacillus* sp. BioSol021 strain to its optimal pH value), as well as dry matter/water content and sugar content, in order to prevent the osmotic shock and substrate inhibition, and to investigate what was the prior dilution of the effluents required before the cultivation (Table 1). Following the upstream procedure, including cultivation media preparation, media and equipment sterilization, as well as inoculum preparation and inoculation, the bioprocess phase included in line monitoring of the key bioprocess parameters (temperature, pH value and DO content) while using the food industry effluents-based media (B1-CW from dairy industry, B2-WFW), which was essential in order to maintain the viability of the producing microorganism cells during the 96 h-long cultivations in the 16 L lab-scale bioreactor. Although corrective actions regarding pH value and dissolved oxygen content weren’t taken during the *Bacillus* sp. BioSol021 cultivations, it is still important to monitor the bioprocess parameters in order to better understand the bioprocess dynamics under the different media conditions. The optimal temperature for growth of the majority of *Bacillus* strains is in the range 25–35 °C; thus, the desired temperature in both B1 and B2 cultivations was set to 28 °C. Higher temperatures can quickly have a dramatic effect on cell viability due to reduced or inhibited enzyme activity and/or physical degradation of the cell structure, while lower temperatures can result in a slower cell metabolism. Considering that it is highly important to maintain a constant temperature during the bioreactor cultivation, automatic temperature regulation was performed by the automatic adjustment of the cold water flow rate through the outside bioreactor jacket placed under the bioreactor vessel based on the temperature sensor response, which has been shown as a suitable method for temperature control considering the constant value of temperature during the both B1 and B2 cultivations at 28 °C, as given in Figure 1. The other important bioprocess parameter is pH value. The initial pH value of the cultivation media based on CW and WFW was set to 7.0, being the optimal pH value for *Bacillus* spp. growth. However, during the sterilization, different chemical reactions take place at the increased temperature, resulting in a slightly changed initial pH value of the cultivation media detected using the pH sensor at the beginning of the cultivations (6.4 in the case of CW-based medium and 6.2 in the case of WFW-based medium). The maximal cell concentration in the CW-based cultivation broth was found in the pH value range of 8.5–9.0. A similar optimal pH value range for production and activity of *Bacillus*-derived alkaline proteases could be observed [46]; hence, the potential production of this enzyme class should be further investigated using the CW medium. On the other hand, the optimum pH value for the WFW-based media in terms of microbial growth was between 6 and 6.5, as cells produce CO_2_ and water as they convert glucose into acidic metabolic products. The antimicrobial activity was recorded across the entire range of the measured pH values (B1: 6.4–9.1; B2: 5.9−7.0). The pH value of the cultivation medium has a significant effect on cell membrane function and morphology, activity of metabolic enzymes and nutrients uptake; therefore, influencing microbial growth and secondary metabolite biosynthesis [47]. Throughout the cultivation of the *Bacillus* sp. BioSol021, while using the CW-based medium, the level of dissolved oxygen (DO) measured as partial oxygen pressure in the cultivation broth (pO_2_) decreased to 50% after 36 h of cultivation and remained in the range of 45–65% until the end of the cultivation. On the other hand, pO_2_ values in the WFW-based medium were constantly decreasing and, at the end of the 96 h-cultivation, it was around 60%. The oxygen requirements of the aerobic microorganisms depend on the type and intensity of metabolic processes requiring oxygen as the final electron acceptor. Therefore, the availability of dissolved oxygen in the cultivation medium could be a limiting factor for metabolic activity. The agitation speed of the cultivation medium, further to the aeration rate itself and the temperature with the effects described by the Henry’s law, also affects the dissolved oxygen levels and thereby stimulates or destimulates various metabolic pathways [48]. Therefore, it is very important to optimize the cultivation conditions in terms of temperature, agitation speed and aeration rate in order to maximize the desired metabolic output in terms of biomass or metabolites content. However, it is also important to consider cost-benefit analysis in terms of energy demands for maintaining the optimized values of the bioprocess variables in order to minimize the energy-related footprint of the production process. 

### 3.2. Microbial Growth Kinetics

**Figure 2:**
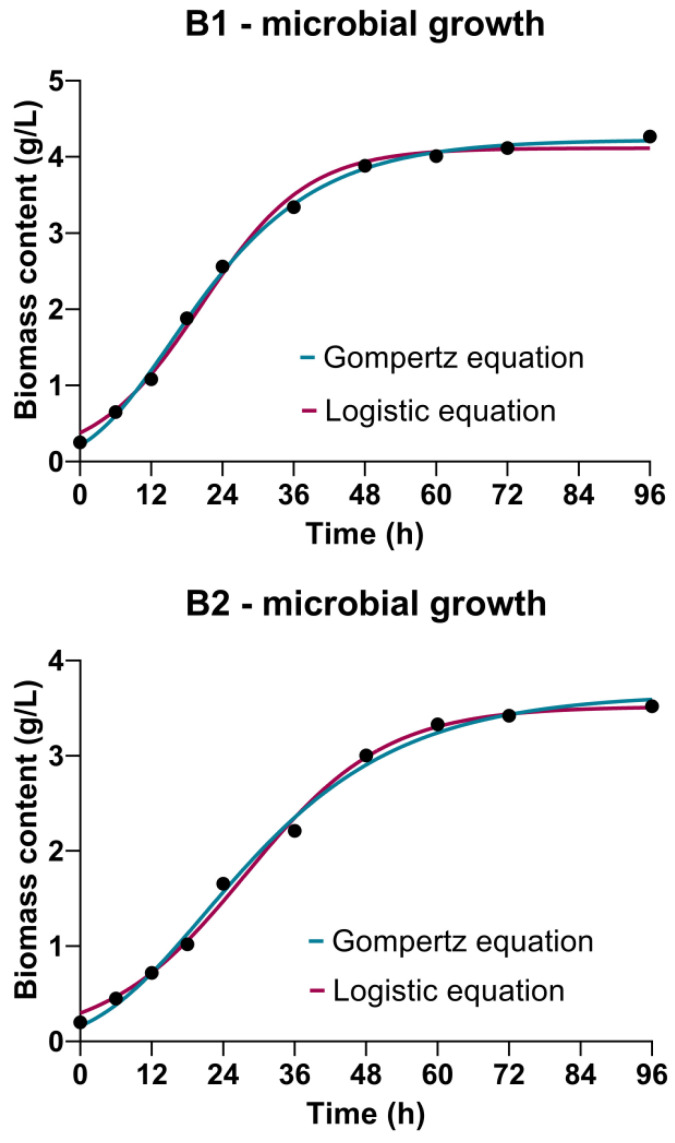
Bioprocess kinetics in terms of *Bacillus* sp. BioSol021 growth during cultivations in a 16 L bioreactor using the food industry effluents-based media: B1—cheese whey from dairy industry, B2—winery flotation wastewater.

Cultivations of *Bacillus* sp. BioSol021 show the conventional bacterial growth pattern using both the CW-based medium containing lactose as the primary carbon source and WFW-based medium containing glucose and fructose as the main carbon sources (Figure 2). It could be observed that the initial biomass concentration of the producing microorganism *Bacillus* sp. BioSol021 was in the narrow range of 0.20–0.25 g/L, indicating the appropriate inoculum preparation procedure resulting in the consistent initial biomass content. The near absence of the lag phase could be noticed in both cases, which could be understood by the presence of the fermentable mono- and disaccharides in both effluents-based cultivation media. The exponential growth phase could be observed until approximately 48 h of the producing microorganism cultivation using both investigated media, followed by the stationary growth phase with slight changes in the biomass content until the end of the cultivation. The absence of the death phase suggests the presence of nutrients that could be further used by the production microorganism *Bacillus* sp. BioSol021, even at the end of the 96 h-cultivation. The final biomass content achieved in the case of the CW medium was 4.27 g/L compared to a biomass content of 3.52 g/L achieved using the WFW-based medium (Figure 2), suggesting that the CW medium supports the growth pattern of *Bacillus* sp. BioSol021 more suitably in terms of the present nutrients. This could be explained by the nutritionally rich content of CW from the microbial point of view, considering the presence of possible nitrogen sources in CW as one of the main nutrients required for the microbial protein and DNA synthesis as microbial biomass components. CW protein content could be in a range of 1.4–8.0 g/L [49], qualifying this byproduct as a valuable source of CW proteins being already isolated as a protein food supplement. However, it is also important to emphasize the significant presence of hydrolyzed proteins, peptides and free amino acids suitable for facilitated microbial consumption in this dairy industry effluent. Due to a suitable nitrogen content profile, CW could be used for microbial growth/metabolite synthesis without additional nitrogen supplementation [50]. On the other hand, cheese whey is also a good source of phosphorus for microbial growth, a with total phosphorus content in the range of 0.12–0.54 g/L [49,51], usually in the form of calcium phosphates. 

The Gompertz equation and the logistic equation were used to fit bacterial growth in terms of *Bacillus* sp. BioSol021 biomass content, but also to determine the main kinetic parameters related to microbial growth, being among the most employed bacterial growth models in predictive microbiology. Both models are classified in the Richard’s family of three-parameter sigmoidal growth models [52], providing information about the initial biomass content *X_0_*, maximal biomass content *X_max_* and maximal specific growth rate *µ*. In order to assess the goodness-of-fit of the experimental data using the selected equations, determination coefficient *R^2^* is given in Table 2 along with each kinetic model. As can be seen in Table 2, the logistic equation predicts slightly higher values of maximal specific growth rate and the initial biomass content, while higher value of the maximal biomass content is predicted by the Gompertz equation in both cultivations, i.e., using the CW and WFW-based media. In the case of B1, i.e., cultivation using the CW medium, the Gompertz equation showed the better prediction ability considering the higher value of *R^2^* (0.9984) compared to logistic equation (0.9954), which was also confirmed with the closer alignment between the model predicted value of the initial biomass content by the Gompertz equation (0.2109 g/L) and the experimental value (0.252 g/L), as well as between the predicted value of the maximal biomass content (4.223 g/L) and the corresponding experimental value (4.267 g/L). In the case of B2, i.e., WFW-based medium used in cultivation of *Bacillus* sp. BioSol021, a slightly higher value of *R^2^* (0.9958) could be observed for the logistic equation compared to the Gompertz equation (0.9956), although the Gompertz equation predicted a closer value of the initial biomass content (0.1606 g/L) to the experimental value (0.2 g/L) compared to the logistic equation (0.2971 g/L). The contrary situation could be observed in the case of maximal biomass concentration, where only a small difference between the value predicted by the logistic equation (3.516 g/L) and the experimental value (3.52 g/L) was noticed. Considering the very high values of the determination coefficient, it could be concluded that both equations could be successfully applied to describe the growth kinetics of *Bacillus* sp. BioSol021 in the media based on CW and WFW. It is also highly important to notice the higher values of maximal specific growth rate predicted by both equations in the case of B1, i.e., cultivation of the producing microorganism using the CW medium, which was in accordance with the higher value of biomass content achieved at the end of the cultivation in comparison to the WFW-based medium, as previously discussed.

### 3.3. Sugar Substrate Consumption Kinetics

**Figure 3:**
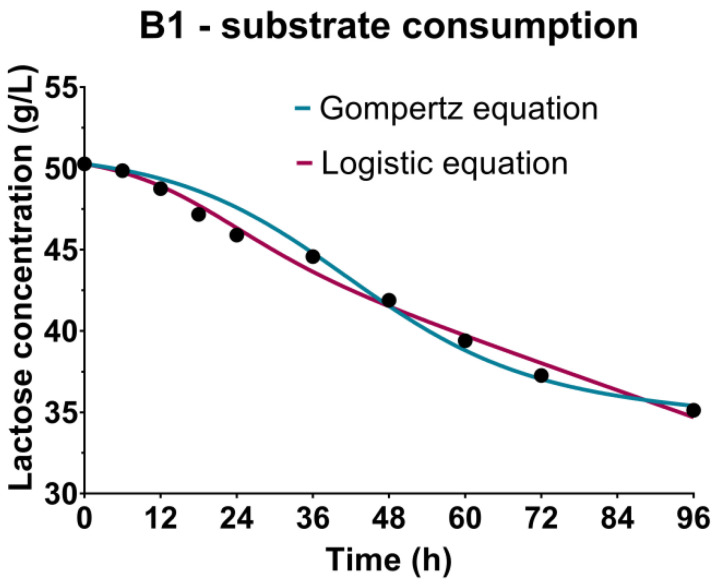

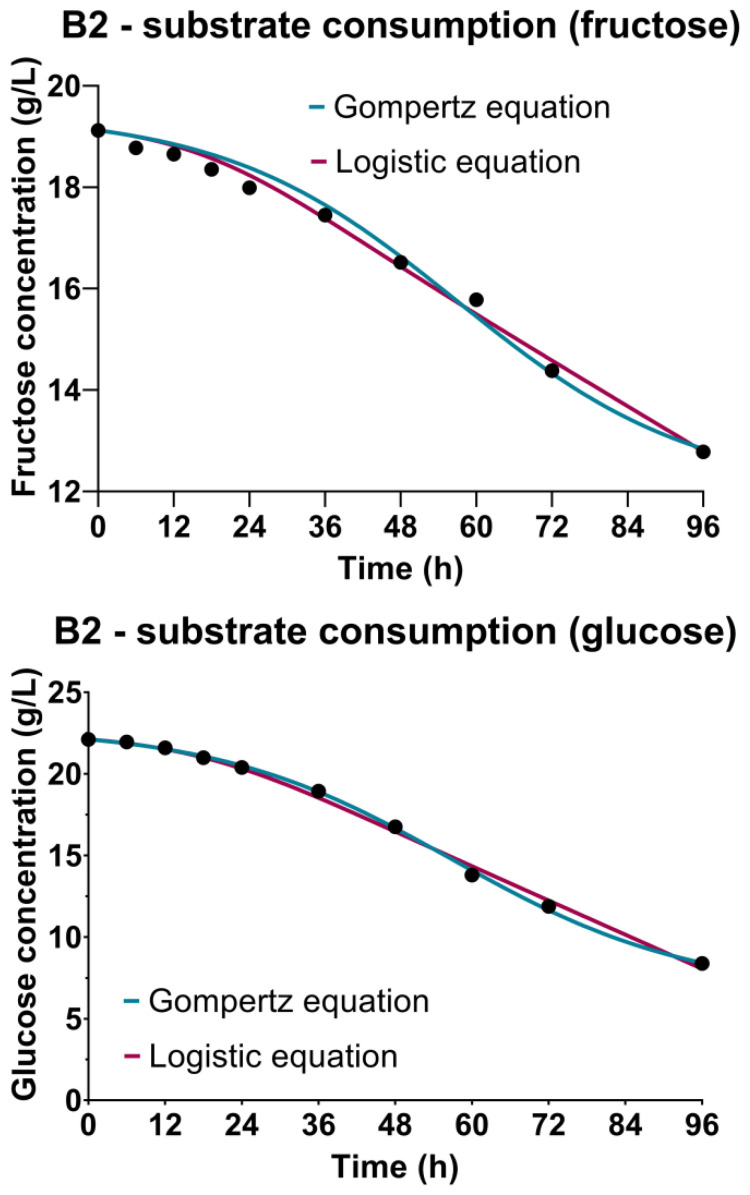
Bioprocess kinetics in terms of sugar substrate consumption during the *Bacillus* sp. BioSol021 cultivations in a 16 L bioreactor using the food industry effluents-based media: B1—cheese whey from dairy industry, B2—winery flotation wastewater.

The changes in substrate content during cultivations were monitored by the determination of sugar sources concentrations, including lactose (Figure 3-B1) as the predominant component of the CW-based medium, and fructose and glucose (Figure 3-B2) present in the WFW-based medium. The continuous decrease of concentrations of carbon sources during cultivations, as observed in Figure 3, can be explained by the nutritive requirements of producing microorganism *Bacillus* sp. BioSol021 for the multiplication, growth and metabolic activity of cells. The first phase of the cultivation was related to the intensified consumption of lactose, fructose and glucose as a consequence of the exponential growth characterized by the highest rate of cell multiplication that lasted approximately 48 h in both experimental sets. The further decrease of the carbon sources after the second day of cultivation, when the producing microorganism entered the stationary growth phase, indicated the metabolic activity of the producing microorganism, which was in accordance with the results obtained for surfactin quantification. The assimilation of carbon sources by the producing microorganism *Bacillus* sp. BioSol021 resulted in decreased residual concentrations of lactose, glucose and fructose in the cultivation broth at the end of the cultivation, with the consumption rates 30%, 70% and 32%, respectively. Previous studies have shown that the majority of *Bacillus* strains prefer glucose as the easily fermentable carbon source for biomass growth and lipopeptide production, with a suggested initial glucose concentration in the cultivation medium in the range of 20–40 g/L [53,54]. Lowering the content of carbon sources, as the main contaminants of waste-based media, is significant from the point of view of ecological efficiency of the created bioprocess solution, considering the positive impact on reduction in environmental burden. In addition to the sustainability aspect, it is important to emphasize economic savings as a consequence of lowering the demands for water utilization in the medium preparation phase [55]. In the particular cases, for CW-based medium, 100% water consumption reduction was achieved as the pure whey was used as the cultivation medium, while the WFW had to be diluted to avoid substrate inhibition due to very high initial sugar concentrations; thus, the savings in this case in terms of water consumption were 25%. The multiple beneficial solution supports the idea of merging different industrial branches within the industrial symbiosis approach that defines the utilization of wastes or by-products of one industry as the raw materials for another [56]. The potential of industrial-level production and the commercialization of value-added biological products is perceived as an important circle in the industrial symbiosis chain through building a sustainable network of energy and materials flow [57]. The biotechnological solutions including the production of microbial biopesticides as products with significant market value are recognized as an important factor in terms of designing the industrial processes with respect to the sustainable development concepts [58]. 

The sugar substrate consumption kinetics were studied by performing regression analysis and numerical optimization from the experimental data, and generating the models using the Luedeking-Piret equation based on the microbial growth kinetic parameters determined by the Gompertz and the logistic equation (Table 2). The requirements of the microorganisms regarding the carbon source could be observed from the perspective of the nutritional requirements necessary for reproduction and growth, but also for the purpose of cell maintenance, explained by non-growth activities such as macromolecules turnover, reparation of damaged cellular components, osmoregulation and cell mobility [59,60]. The coefficient indicating the contribution of substrate consumption to cell multiplication and growth, directly proportional to the growth rate, is marked as *α* (g_substrate_/g_biomass_), and the other one *β* (1/h), describes the substrate consumption to the maintenance of the cells during the stationary growth phase, proportionally to the biomass concentration. Better model fitness in the case of B1, i.e., CW-based medium used in cultivation of *Bacillus* sp. BioSol021, was observed when using the microbial growth parameters obtained by the logistic equation, taking into account a higher *R^2^* value (0.9908) compared to the Gompertz equation (0.9771) (Table 2). The parameters of the Luedeking-Piret model indicated higher values of *α* coefficients; 3.470 g_substrate_/g_biomass_ and 1.410 g_substrate_/g_biomass_ when using kinetic growth parameters defined by the Gompertz and the logistic equations, respectively, indicating a more significant influence of the substrate consumption on producing microorganism cell growth. Lower levels of *β* coefficients are observed for both models; in the case of kinetic growth parameters from the Gompertz equation, 0.0053 1/h, and in the case of the logistic equation, 0.0336 1/h, implying lower nutrient requirements for cell maintenance during the stationary cultivation phase. In the case of the second experimental set, B2, performed using WFW-based medium, similar *R^2^* values indicated excellent model fitness for both models, and for both carbon sources, fructose and glucose. A slight advantage for the mathematical description of glucose consumption in the WFW-based medium could be given to the growth parameters obtained by the Gompertz equation (*R^2^* 0.9991) compared to the logistic equation (*R^2^* 0.9961). The opposite could be said for fructose consumption, with *R^2^* values of 0.9920 and 0.9874 for the parameters obtained by the logistic and the Gompertz equations, respectively. Regarding sugar source consumption for reproduction and non-growth activities, it could be concluded that according to the Luedeking-Piret equation parameters, glucose was the preferred carbon source, contributing the most to the growth of the producing microorganism with the value 4.331 g_substrate_/g_biomass_. The growth coefficient value for fructose obtained using the Gompetz equation’s growth parameters was 1.974 g_substrate_/g_biomass_, while the coefficients describing non-growth related activities were 0.0010 1/h and 0.0015 1/h for fructose and glucose, respectively. The use of the growth parameters of the logistic equation confirms the greatest contribution of glucose to the reproduction of the producing microorganism, with a growth-related coefficient value of 0.7416 g_substrate_/g_biomass_, compared to contributions to cell maintenance described by the *β* coefficients with values 0.0211 1/h for fructose and 0.494 1/h for glucose (Table 2).

### 3.4. Surfactin Production Kinetics

When it comes to surfactin quantification, for the rest of the lipopeptide compounds, the most accurate method is HPLC-MS quantification. However, several other methods were proposed in order to simultaneously decrease analysis cost and time, achieving similar accuracy. For example, the CPC-FL (cetylpyridinium chloride-fluorescein) method was suggested by Heuson et al. [61], based on fluorescence measurements after the release of fluorescein from the CPC-FL complex when surfactin was added to the mixture. A similar CPC-BTB method for colorimetric detection and quantification of surfactin was proposed by Yang et al. [42], with the difference of applying bromothymol blue as a cost-effective option instead of fluorescein. One of the simplified methods for lipopeptides quantification proposed by Ong et Wu [62] could be employed to colorimetrically detect and quantify the overall lipopeptide content in a bacterial cultivation broth or supernatant sample by only using bromothymol blue. In this study, the colorimetric method for surfactin quantification proposed by Yang et al. (2015) [42] was used, considering that a surfactin-specific method is suitable for fast and reliable offline bioprocess monitoring in terms of surfactin quantification, resulting in the increased cost-effectiveness of the bioprocess-related analyses in comparison with other suggested methods. Supernatant samples obtained by the cultivation broths centrifugation to remove *Bacillus* sp. BioSol021 biomass were used for surfactin quantification, considering its extracellularly production. 

Considering the nutrient broth was used as the medium in the inoculum preparation phase, the initial surfactin concentration in the cultivation broths based on both CW and WFW could be explained by its introduction in the cultivation broth with the producing microorganism’s inoculum. As can be seen in Figure 3, a slight increase in the surfactin concentration could be observed until the cultivation period between 36 h and 48 h, corresponding to the exponential growth phase. After that, a sharper increase in surfactin concentration was detected during the stationary growth phase, considering that its biosynthesis is predominantly related to the secondary metabolism. The highest achieved surfactin concentration in the CW medium was 1.54 g/L, while in the WFW-based medium it was 2.65 g/L. Considering the higher dry matter content in the WFW, which could be observed as colloidal turbidity, the presence of suspended particles could be beneficial when it comes to surfactin synthesis. Yeh et al. [63] investigated the addition of different solid carriers to promote cell growth, biofilm formation and surfactin synthesis, resulting in a 36-fold increase in surfactin yield when adding activated carbon as the solid carrier. The difference in the surfactin concentration could be also attributed to a greater availability of easily fermentable monosaccharides in the WFW-based medium compared to CW-based medium, with an emphasis on glucose as the usual carbon source used in the media for surfactin synthesis [14]. However, semisynthetic media suggested for surfactin biosynthesis are one of major reasons for its high market price and being less competitive compared to petrochemically derived surfactants, considering that cultivation medium cost makes up to 30–50% of the overall surfactin production cost [64]. Hence, different alternative media, mostly based on agroindustrial residues, have been suggested as possible routes to decrease the surfactin production cost. Raw glycerol from the biodiesel industry has been suggested as a medium basis for surfactin production, resulting in a surfactin yield in the range of 71–286 mg/L [65,66]. Brewery wastewater, as a surfactin production medium, resulted in a surfactin yield of 6.6 mg/L [67], while distillers’ grains applied as a substrate gave a better result, with a yield of 1.04 g/L [13]. Medium based on rehydrated whey powder has yielded significantly lower surfactin concentrations (0.18–0.24 g/L) [68] compared to the results of this study, while comparable results in terms of surfactin yield (up to 3 g/L) were achieved by applying cassava wastewater as a cultivation medium [69,70,71].

**Figure 4:**
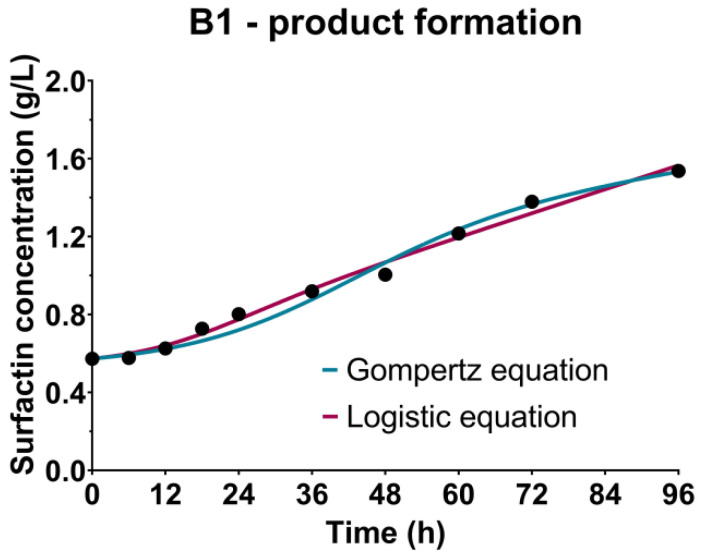

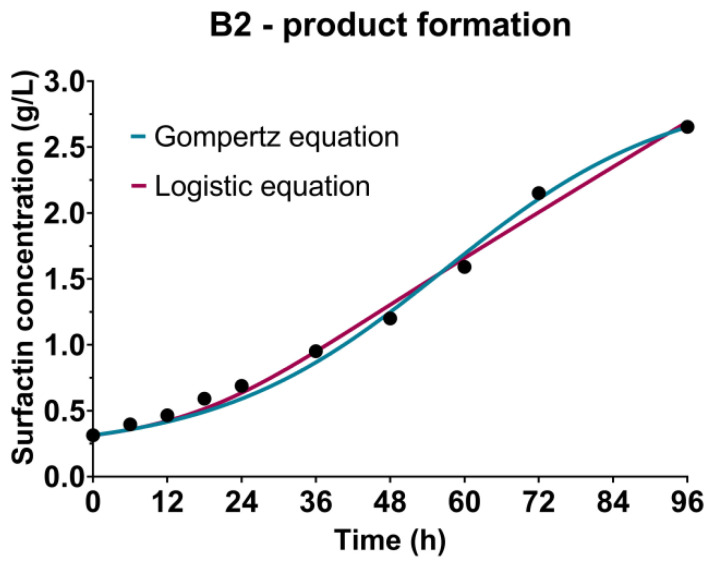
Bioprocess kinetics in terms of surfactin production during the *Bacillus* sp. BioSol021 cultivations in a 16 L bioreactor using the food industry effluents-based media: B1—cheese whey from dairy industry, B2—winery flotation wastewater.

The kinetic growth parameters previously determined using the Gompertz and the logistic equation were used as a basis for product - surfactin formation kinetics, described by the Luedeking-Piret equation. As could be seen in the case of B1-cultivation based on the CW medium (Table 2, Figure 4), higher values of both coefficients of the Luedeking-Piret equation were predicted by using the kinetic growth parameters obtained by the logistic equation, also showing higher *R^2^* value (0.9897) compared to the Gompertz equation (0.9841). On the other hand, a better fitting of the experimental data in the case of B2 (WFW-based medium) was achieved using the kinetic growth parameters from the Gompertz equation (Table 2, Figure 4). According to Gaden’s classification of the formation mode of the product, based on the values of the product formation constants *γ* and *δ* [72], surfactin biosynthesis was partially related to the growth of *Bacillus* sp. BioSol021 as it had been classified as Class II, where γ ≠ 0 and δ ≠ 0, which can also be observed by comparing the experimental data presented in Figure 2 and Figure 4.

### 3.5. Antimicrobial Activity against the Aflatoxigenic Aspergillus flavus Strains

**Figure 5:**
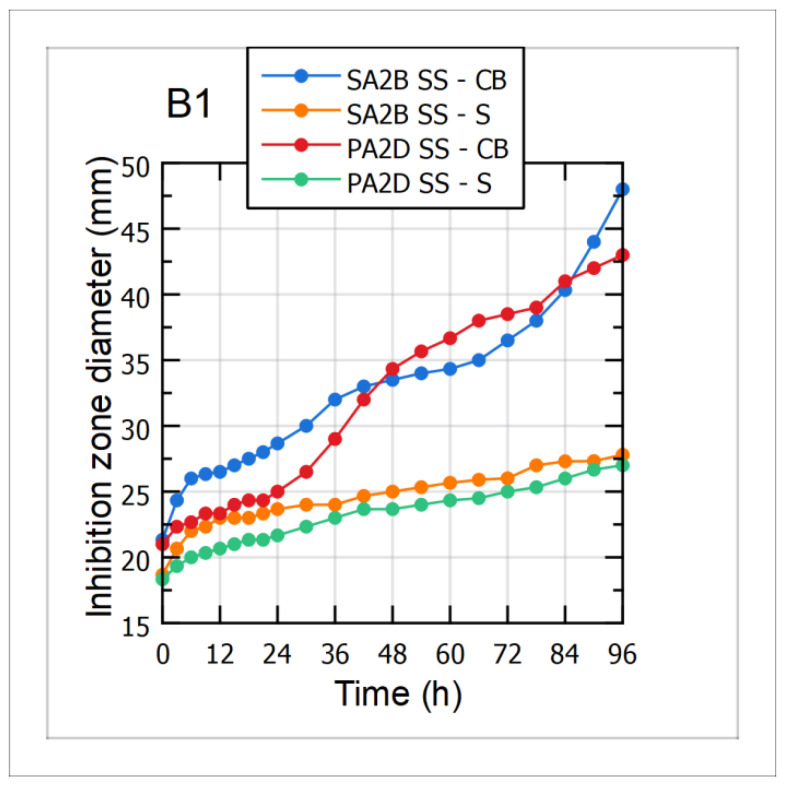

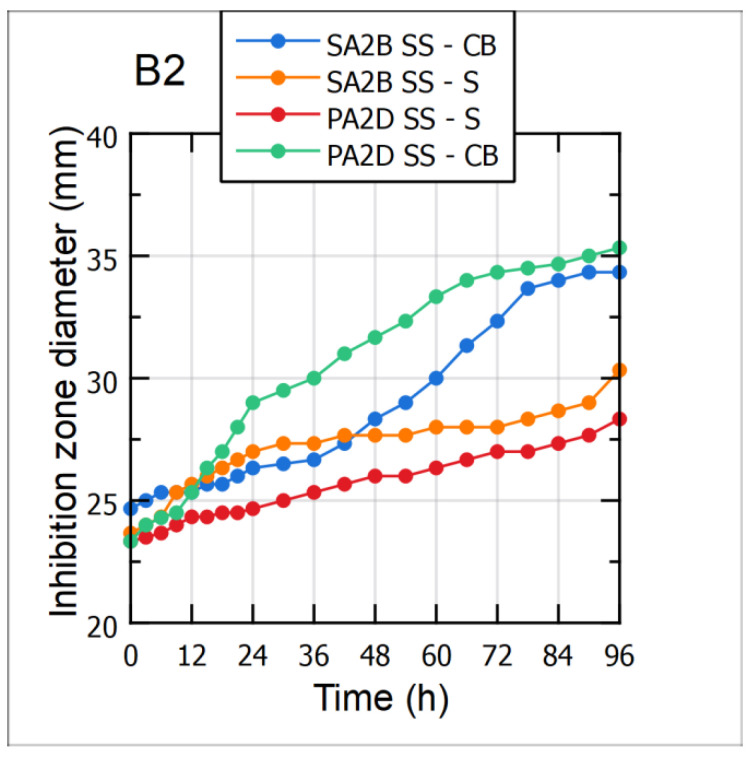
Antimicrobial activity of the *Bacillus* sp. BioSol021-based biocontrol agents against the aflatoxigenic *Aspergillus flavus* strains SA2B SS and PA2D SS using the food industry effluents-based media for the cultivations of the producing microorganism: B1—cheese whey from dairy industry, B2—winery flotation wastewater, CB—cultivation broth, S—supernatant.

Antagonistic activity of cultivation broth (CB) and cell-free supernatant (S) samples during the 96 h long cultivations of the *Bacillus* sp. BioSol021 using CW and WFW-based media was evaluated against the two aflatoxigenic strains, *Aspergillus flavus* PA2D SS and SA2B SS. Mean values of the inhibition zone diameters, as direct indicators of the suppressive effect level, are shown in Figure 5-B1 for the CW-based medium and Figure 5-B2 for the WFW-based medium. As can be observed from the graphical illustrations, the results indicated that higher antagonistic activity was expressed by the cultivation broth samples against both aflatoxigenic strains, implying the dominant role of producing microorganism cells as active components of the cultivation broth samples. The changes in the suppressive effect of the CB samples during the cultivation were in accordance with the increase of the producing microorganisms’ cell concentration and its continuous growth, with the biggest inhibition zone diameters recorded after the fourth day of the observed bioprocesses. Regarding the level of antimicrobial activity, it could be seen that *Bacillus* sp. BioSol021-CB samples, in the case of the CW medium, showed higher inhibitory activity against the tested aflatoxigenic strains compared to the results obtained for the WFW medium. The highest antimicrobial activity, with the inhibition zone diameter of almost 50 mm, was obtained in the case of the *Bacillus* sp. BioSol021-CB sample against the *Aspergillus flavus* SA2B SS. Slightly lower susceptibility of the tested aflatoxigenic strains was observed when cell-free supernatant samples from the both experimental sets were used. On the other hand, the presence of the inhibition effect of cell-free supernatant samples could be explained by antibiosis as an important biocontrol mechanism, including the confirmed ability for biosynthesis of surfactin. The increase in inhibition zone diameters for the cell-free supernatant samples during cultivation is in accordance with the results obtained for surfactin quantification, implying its contribution to the antagonistic activity. Considering the inhibition zone diameters obtained as results of supernatant activity, the highest suppressive effect was recorded in the case of the CW cell-free supernatant sample obtained at the end of the cultivation and tested against the *Aspergillus flavus* SA2B SS, with the inhibition zone diameter above 30 mm. 

Summarizing the obtained results, it can be concluded that the strongest antagonistic effect against the tested aflatoxigenic fungi could be achieved using the final form of the biocontrol product containing the cultivation broth of the producing microorganism. This explanation is determined by the synergistic effect of multiple mechanisms of action, including the competitive activity of bacterial biomass for space and nutrients, and the antimicrobial activity of the produced metabolites. Regarding surfactin, as the metabolite of interest examined in the present study, it is a lipopeptide mostly recognized by its powerful biosurfactant properties. However, a number of studies have confirmed its significant contribution to the suppression of fungal pathogens, whether it is expressed by its own activity [73,74] or by synergistic effect with other lipopeptides with antifungal activity from iturin and fengycin families [75,76]. Previous studies have also confirmed that the surfactin-producing strain *Bacillus subtilis* BS119 showed a complete inhibitory effect against *Aspergillus flavus*, while the synergistic effect of surfactin and fengycin resulted in stronger suppression of *Aspergillus flavus* R5 [77]. In the study published by Mousivand et al. [78], representatives of the surfactin-producing strains collection of *Bacillus subtilis* were evaluated as efficient antagonistic agents against *Aspergillus flavus*. Additionally, the contribution of surfactin from the perspective of biocontrol is defined by its intense biosurfactant activity and the support in biofilm formation [18]. When it comes to the usual mechanisms of antimicrobial activity expressed by the surfactin lipopeptides, physico-chemical activity is reflected in cell membrane disintegration or osmotic pressure imbalance, followed by the inhibition of protein synthesis and enzyme activity, resulting in the reduced or completely inhibited metabolic activity and reproduction of the phytopathogens [79]. 

From another perspective, favoring the bioprocess design that includes cultivation broth in the final formulation implies the techno-economic feasibility of the proposed biotechnological solution. The absence of complicated and cost-demanding downstream processing procedures is important in the context of not only lowering the overall production costs, but also considering the sustainability and ecological impact of the created technology on the environment. An additional advantage of the product containing cultivation broth comes from the presence of residual nutrients, which could be beneficial for application in agricultural production in terms of soil quality improvement. Further research will include detailed analyses of adequate formulation approaches, with the aim of attaining the maximum activity of the biocontrol agent, as well as an investigation of the potential influence of the created product on soil properties and plant cultivation.

## 4. Conclusions

The results of this study have shown the high potential of CW and WFW to be used as media basis for production of microbial biocontrol agents based on *Bacillus* sp. BioSol021. The CW-based medium showed better results in terms of microbial growth improvement, while a higher concentration of surfactin was synthesized in the WFW-based medium. However, in both cases, the highest biocontrol activity against aflatoxigenic *Aspergillus flavus* strains was achieved when using the complete cultivation broth as the biocontrol agent, suggesting the possibility to reduce the downstream processing cost by the absence of necessity to separate microbial biomass and to further purify the produced antimicrobial metabolites. Further techno-economic analysis is required to assess the industrial viability of the proposed valorization routes, with a possibility for further bioprocess optimization in terms of cultivation conditions in order to maximize the desired output regarding biocontrol activity, as well as to maximize the bioprocess energetic cost-effectiveness. Further research will also include *in planta* tests in semi-controlled and field conditions to investigate real-life application and efficiency of the produced biocontrol agents, as well as their effects on the soil ecosystem.

## Figures and Tables

**Table 1:** Quality parameters of the food industry effluents used as media basis for production of microbial biocontrol agents.

Parameter/Effluent	pH Value	Dry Matter/Water Content (%, *w*/*v*)	Sugar Content (g/L)
CW	4.90	5.81/94.19	50.28 ^L^
WFW	3.60	17.01/82.99	57.36 ^F^ 66.33 ^G^

CW—cheese whey, WFW—winery flotation wastewater, L—lactose, F—fructose, G—glucose.

**Table 2:** Kinetic parameters related to microbial growth, substrate consumption and product formation during the cultivations of *Bacillus* sp. BioSol021 in a 16 L bioreactor using the food industry effluents-based media: B1—cheese whey from dairy industry, B2—winery flotation wastewater.

	B1				B2			
	Gompertz Equation	*R* *^2^*	Logistic Equation	*R* *^2^*	Gompertz Equation	*R* *^2^*	Logistic Equation	*R* *^2^*
**Microbial growth**								
*X_0_* (g/L)	0.2109	0.9984	0.3754	0.9954	0.1606	0.9956	0.2971	0.9958
*X_max_* (g/L)	4.223		4.113		3.652		3.516	
*µ* (1/h)	0.0722		0.1123		0.0544		0.0856	
**Substrate consumption**								
*α* (g_substrate_/g_biomass_)	3.470 ^L^	0.9771 ^L^	1.410 ^L^	0.9908 ^L^	1.974 ^F^ 4.331 ^G^	0.9874 ^F^ 0.9991 ^G^	0.4179 ^F^ 0.7416 ^G^	0.9920 ^F^ 0.9961 ^G^
*β* (1/h)	0.0053 ^L^		0.0336 ^L^		0.0010 ^F^ 0.0015 ^G^		0.0211 ^F^ 0.0494 ^G^	
**Product formation**								
*γ* (g_substrate_/g_biomass_)	0.1769	0.9841	0.6137	0.9897	0.7466	0.9923	0.1453	0.9913
*δ* (1/h)	0.0011		0.0025		0.0001		0.0081	

L—lactose, G—glucose, F—fructose.

## Data Availability

No new data were created or analyzed in this study. Data sharing is not applicable to this article.
